# FOXA3, a Negative Regulator of Nur77 Expression and Activity in Testicular Steroidogenesis

**DOI:** 10.1155/2021/6619447

**Published:** 2021-03-03

**Authors:** Hansle Kim, Sudeep Kumar, Keesook Lee

**Affiliations:** School of Biological Sciences and Technology, Chonnam National University, Gwangju, Republic of Korea

## Abstract

Biosynthesis of testosterone occurs mainly in the testicular Leydig cells. Nur77, an orphan nuclear receptor that is expressed in response to the luteinizing hormone/cyclic adenosine monophosphate (LH/cAMP) signaling pathway, is one of the key factors that regulate steroidogenesis in Leydig cells. The function of Nur77 is modulated through interaction with other proteins. FOXA3, a transcription factor that is crucial for male fertility, is also expressed in Leydig cells. Here, we sought to elucidate the role of FOXA3 in testicular steroidogenesis by focusing on its interaction with Nur77. LH/cAMP signaling induces the onset of steroidogenesis in Leydig cells but has a repressive effect on the expression of FOXA3. Overexpression of FOXA3 in MA-10 Leydig cells repressed cAMP-induced expression of *Nur77* and its target steroidogenic genes (*StAR*, *P450c17*, and *Hsd3β*). Furthermore, FOXA3 suppressed Nur77 transactivation of the promoter of steroidogenic genes. In mouse primary Leydig cells, adenovirus-mediated overexpression of FOXA3 had similar effects and resulted in decreased production of testosterone. Taken together, these results suggest the role of FOXA3 in the regulation of steroidogenic genes in Leydig cells and fine-tuning steroidogenesis in the testis.

## 1. Introduction

Leydig cells in the testis produce hormones and growth factors that are essential for the development and proper functioning of the male reproductive system [1, 2]. Testosterone, a pivotal male hormone, is mainly produced in testicular Leydig cells, and its production is regulated by steroidogenic proteins, such as StAR, P450c17, and HSD3B. Among the various transcription factors that regulate the expression of steroidogenic genes, Nur77 plays a major role [3, 4]. Luteinizing hormone (LH), a regulator of steroidogenesis, induces the expression of Nur77 in Leydig cells [5], which in turn regulates the expression of steroidogenic genes such as steroid 21-hydroxylase, 20*α*-hydroxysteroid dehydrogenase, and P450c17 [3, 6, 7]. Nur77 directly binds to defined regions in the promoter of rat P450c17, mouse StAR, and human HSD3B2 and regulates their expression [3, 4, 8]. The expression and activity of Nur77 are modulated by other cellular proteins, such as CREB, DAX1, and c-JUN, and nuclear receptors, such as ERR*γ*, AR, GR, and NOR1 [9–12].

Members of the forkhead box A (FOXA) subfamily including FOXA1, FOXA2, and FOXA3 are mainly known for their roles in the liver [13, 14]. These genes have multiple roles in mammalian organ development [15]. Among the *Foxa* subfamily of transcription factors, only *Foxa3* is expressed in the testis, mainly in Leydig and postmeiotic germ cells [16, 17]. *Foxa3* mutant mice showed abnormal testicular development, could not support spermatogenesis, and exhibited increased apoptosis in the germinal epithelium [16]. The expression of FOXA3 is most abundant in the nucleus of adult Leydig cells and to a lesser extent in some Sertoli and peritubular cells [18]. FOXA3 regulates the expression of platelet-derived growth factor receptor-*α* (PDGFR*-α*) in Leydig cells [18]. However, the function of FOXA3, an important transcription factor associated with male fertility, is poorly understood in testicular Leydig cells.

In the current study, we found that unlike Nur77 expression, the expression of *Foxa3* is repressed during cAMP-induced steroidogenesis in Leydig cells. This led us to investigate the effect of FOXA3 on Nur77 expression and transactivation and also on testicular steroidogenesis. FOXA3 directly represses cAMP-induced expression of Nur77 and inhibits Nur77-mediated transactivation of its target steroidogenic genes in Leydig cells. The repression of steroidogenic genes results in reduced production of testosterone. Altogether, these results suggest a modulatory effect of FOXA3 in testicular steroidogenesis.

## 2. Materials and Methods

### 2.1. Animals

C57BL/6 mice (6-week-old) were purchased from a commercial supplier (Dae-han Laboratories, Daejeon, Korea) and maintained in a 12 : 12 h light-dark cycle with food and water ad libitum. The mice were sacrificed by CO_2_ asphyxiation, following which their testes were dissected. The animals were treated ethically according to the National Institutes of Health standards. All animal procedures were approved by the Institutional Animal Care and Use Committee (AICUC) of Chonnam National University (Permit Number: 2012-44).

### 2.2. Plasmids and Chemicals

pcDNA-Nur77, reporter plasmid Nur77 promoter (−413/−34)-luc, NurRE-luc, mouse StAR (−1533/+34)-luc, mouse P450c17 (−1040/+3)-luc, mouse 3*β*-HSD (−4700/+40)-luc, and CRE-luc have been described previously [5, 9]. pcDNA3-*Foxa3* and adenoviral (Ad) vectors encoding GFP only (Ad-only) and GFP with *Foxa3* (Ad-*Foxa3*) have been described previously [19]. 8Br-cAMP was purchased from Sigma-Aldrich (St Louis, MO, USA).

### 2.3. Cell Culture, Transient Transfection, and Luciferase Assay

MA-10 cells (kind gift from Dr. Mario Ascoli; University of Iowa, IA, USA) [20] were maintained in Roswell Park Memorial Institute 1640 (RPMI 1640) medium (Hyclone, UT, USA), supplemented with 25 mМ HEPES, 2 mМ L-glutamine, 15% horse serum, and antibiotics. All cells were cultured at 37°C under an atmosphere of 5% CO_2_. For luciferase assay, MA-10 cells were plated in media containing 5% charcoal-stripped serum 24 h prior to transfection and transfected with expression vectors, a reporter gene, and the control *lacZ* expression plasmid, pSV-*β*-gal (Promega Corporation, WI, USA). Transfection was performed using Lipofectamine 2000 (Life Technologies Corporation, Carlsbad, CA, USA) according to the manufacturer's instructions. The total amount of transfected DNA was kept constant by adding pcDNA3 empty vector. Luciferase and *β*-galactosidase activities were assayed as described previously [21]. After 48 h, the cells were harvested and lysed in cell lysis buffer (0.2 М Tris (pH 8.0) and 0.1% Triton X-100). Cell lysates were used to measure the luciferase activity. The luciferase activity was normalized to *lacZ* expression.

### 2.4. Isolation of Primary Leydig Cells

Primary mouse Leydig cells were isolated as described previously [10]. Briefly, decapsulated testicular cells were dispersed with collagenase type І (0.25 mg/mL, Life technologies Corporation, Grand Island, NY, USA) in M199/EBSS medium. The testicular cells were maintained at 25°C for 20 min with gentle shaking and tapped every 5 min to disperse the testicular tubules. Medium containing the cells was collected and filtered with a 40 *μ*m cell strainer (BD Biosciences, San Jose, CA, USA). The collected cells were washed twice with M199/EBSS medium and pelleted by centrifugation. Finally, the cells were plated in RPMI 1640 medium (containing 25 mM HEPES) supplemented with 15% horse serum and antibiotics.

### 2.5. Radioimmunoassay (RIA)

To measure testosterone levels, serum-free culture medium was collected from primary mouse Leydig cells that were infected with Ad-only or Ad-FOXA3 and treated with cAMP for 48 h. Medium was separated by centrifugation at 10,000 × g for 5 min at 4°C and stored at −70°C until testosterone assay. Testosterone concentration was measured using RIA as described previously [22]. The interassay and intraassay coefficients of variation for the testosterone estimation were 8.7% and 9.3%, respectively [23].

### 2.6. Quantitative Real-Time Polymerase Chain Reaction (qRT-PCR)

Reverse transcription reactions were performed with 2 *μ*g of total RNA using M-MLV reverse transcriptase (Promega, Madison, WI, USA). Quantitative PCR was performed with TOPreal qPCR 2X PreMIX, SYBR green with high ROX (Enzynomics, Daejeon, Republic of Korea) using the StepOnePlus^TM^ Real-Time PCR System (Applied Biosystems, Carlsbad, CA) according to the manufacturer's instructions. The primer sequences of the genes are listed in the Supplementary Table 1.

### 2.7. Statistical Analysis

To identify significant differences, data were analyzed using Graph Pad Prism version 5.0. Single comparisons between two experimental groups were performed using the unpaired Student's *t*-test. The data are presented as the mean ± SEM of at least three independent experiments. *P* < 0.05 was considered statistically significant.

## 3. Results

### 3.1. Decreased Expression of *Foxa3* mRNA in cAMP-Induced MA-10 Leydig Cells

**Figure 1:**
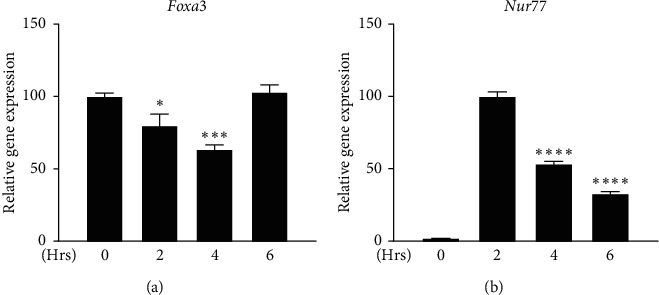
Expression of *Foxa3* mRNA decreases during cAMP induction in MA-10 Leydig cells. Time-dependent expression of *Foxa3* decreases up to 4 hours and is restored at 6 h (A), whereas the expression of Nur77 is strongly induced at 2 h and then decreases (B) in cAMP-induced MA-10 cells. *Nur77* expression at 2 h and *Foxa3* expression at 0 h were considered 100% for relative comparison. The gene expression level was normalized to that of *β*-actin. For statistical significance, the *t*-test has been performed. ^*∗*^*P* < 0.05; ^*∗∗*^*P* < 0.01; ^*∗∗∗*^*P* < 0.001.

Leydig cells start steroidogenesis by expressing steroidogenic genes following induction by LH/cAMP signaling. To investigate the responsiveness of FOXA3 to LH/cAMP signaling, we stimulated MA-10 cells with cAMP for different time periods and compared it with the expression of *Nur77*. The expression of *Foxa3* was repressed at 2 h, further repressed at 4 h, and then restored at 6 h point of cAMP stimulation (Figure 1(a)). In contrast, *Nur77* expression was strongly induced at 2 h and decreased thereafter (Figure 1(b)).

### 3.2. FOXA3 Represses cAMP-Induced Promoter Activity of Nur77 and Its Target Steroidogenic Genes

**Figure 2:**
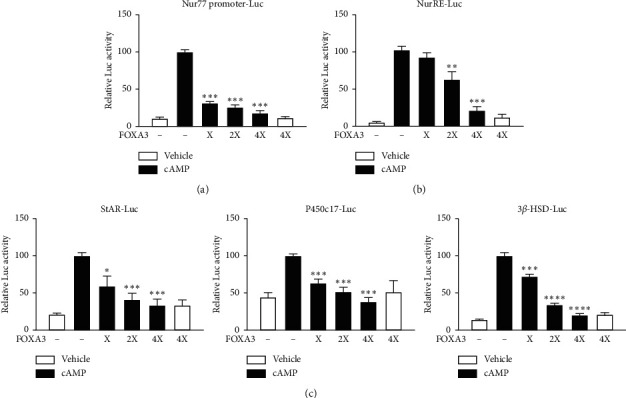
FOXA3 represses cAMP-induced promoter activity of Nur77 and its target steroidogenic genes. FOXA3 strongly represses cAMP-induced promoter activity of Nur77 (A) and Nur77 responsive element Nur-RE (B) in a dose-dependent manner. Nur77 target steroidogenic gene promoters are also repressed by overexpression of FOXA3 in cAMP-stimulated MA-10 Leydig cells (C). Cells were transfected with different reporter constructs, SV-40 *β*-gal, and indicated concentration of FOXA3 (X represents 100 ng of the expression construct and 2X and 4X represent the fold amounts of (X)) for 48 h that included overnight cAMP induction (200 *μ*M). Luciferase activity was normalized to *β*-galactosidase activity. The cAMP-induced controls were considered 100% for comparison, and the *t*-test was performed to analyze statistical significance. ^*∗*^*P* < 0.05; ^*∗∗*^*P* < 0.01; ^*∗∗∗*^*P* < 0.001; ^*∗∗∗∗*^*P* < 0.0001.

The opposite expression pattern of *Foxa3* and *Nur77* in response to cAMP stimulation at 2 h and 4 h in MA-10 Leydig cells (Figure 1) led us to investigate the effect of FOXA3 on Nur77 promoter activity using transient transfection. We found that the cAMP-induced Nur77 promoter activity was repressed by FOXA3 overexpression in MA-10 cells (Figure 2(a)). A promoter containing the Nur77 response element (NurRE-Luc) was also repressed in a dose-dependent manner by FOXA3 (Figure 2(b)). As expected, Nur77 target steroidogenic gene promoters including StAR, P450c17, and 3*β*-HSD were repressed by FOXA3 in a dose-dependent manner (Figure 2(c)). Taken together, FOXA3 suppresses cAMP-induced activity of Nur77 and its target steroidogenic gene promoters.

### 3.3. FOXA3 Inhibits the Transactivation of CREB, Which Regulates Nur77 Promoter Activity

**Figure 3:**
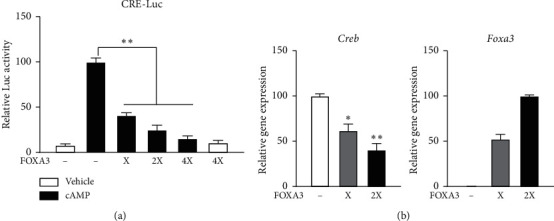
FOXA3 represses cAMP-induced CREB transactivation and expression in MA-10 cells. FOXA3 strongly inhibits cAMP-induced CREB transactivation of a CRE-dependent promoter in MA-10 cells. Cells were transfected with a CRE-Luc reporter, SV-40 *β*-gal, and indicated concentration of FOXA3 (X represents 100 ng of the expression construct) for 48 h that included overnight cAMP induction (200 *μ*M). Luciferase activity was normalized to *β*-galactosidase activity (A). FOXA3 represses CREB expression in MA-10 cells. *Foxa3* and *Creb* expression were quantified using qRT-PCR. The gene expression level was normalized to that of GAPDH. Cells were transfected with indicated concentrations of FOXA3 (X represents 200 ng of the expression construct) for 48 h that included cAMP induction (200 *μ*M) for 4 h (B). Statistical significance was assessed using the *t*-test. ^*∗*^*P* < 0.001; ^*∗∗*^*P* < 0.0001.

We investigated the effect of FOXA3 on the transactivation of CREB, which regulates Nur77 expression upon cAMP induction in Leydig cell [24], using a CRE-Luc reporter. FOXA3 inhibited cAMP-activated CREB transcriptional activity in a dose-dependent manner, repressing the expression of CRE-Luc (Figure 3(a)). Furthermore, overexpression of FOXA3 reduced *Creb* expression in MA-10 cells (Figure 3(b)). These results suggest that FOXA3 represses the cAMP-induced Nur77 promoter through the inhibition of CREB transcriptional activity and expression.

### 3.4. FOXA3 Downregulates the Expression of *Nur77* and Its Target Steroidogenic Genes

**Figure 4:**
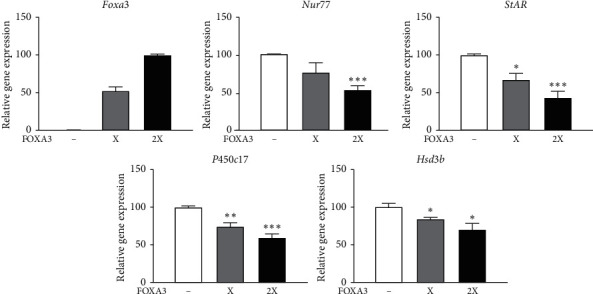
FOXA3 regulates the expression of Nur77 and steroidogenic genes. Expression of Nur77 and steroidogenic genes in MA-10 cells transfected with pcDNA3-only or pcDNA3-FOXA3 (X represents 200 ng of the expression construct). After 44 h of transfection, cells were treated with 200 *μ*M cAMP for 4 h. Gene expression was quantified by qRT-PCR, and the gene expression level was normalized to that of GAPDH. The *t*-test was performed to analyze statistical significance. ^*∗*^*P* < 0.01; ^*∗∗*^*P* < 0.001; ^*∗∗∗*^*P* < 0.0001 compared to cAMP-induced samples.

Overexpression of FOXA3 downregulates the promoter activity of Nur77. Therefore, we investigated the expression of *Nur77* and its target steroidogenic genes following cAMP induction in MA-10 cells. Consistent with the repressed promoter activity found in the promoter-reporter assays, mRNA expression of *Nur77* and its target steroidogenic genes including *StAR*, *P450c17*, and *Hsd3b* were all repressed following overexpression of FOXA3 (Figure 4).

### 3.5. FOXA3 Inhibits Nur77-Mediated Transactivation of Steroidogenic Gene Promoters

**Figure 5:**
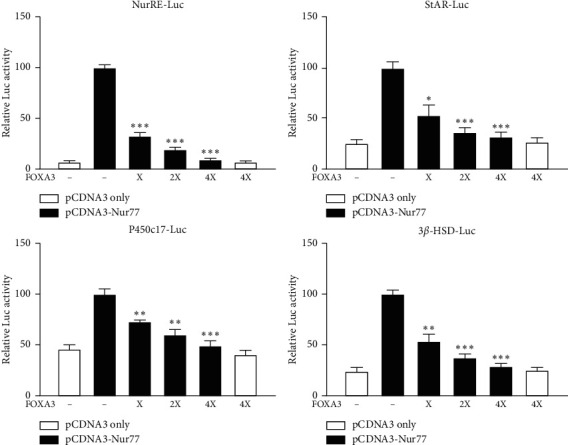
FOXA3 inhibits Nur77-mediated transactivation of steroidogenic gene promoters. FOXA3 represses Nur77-mediated transactivation of steroidogenic gene promoters in a dose-dependent manner in MA-10 cells. Cells were transfected with different reporter constructs, SV-40 *β*-gal, and indicated concentration of FOXA3 (X represents 100 ng of the expression construct) for 48 h Luciferase activity was normalized to *β*-galactosidase activity. The cAMP induced controls were considered 100% for comparison, and the *t*-test was performed to analyze statistical significance. ^*∗*^*P* < 0.01; ^*∗∗*^*P* < 0.001; ^*∗∗∗*^*P* < 0.0001.

FOXA3 represses the expression of Nur77 and consequently inhibits the expression of its target steroidogenic genes (Figure 4). However, we cannot rule out the possibility that FOXA3 directly interferes with Nur77-mediated transactivation of its target genes. To assess this, we performed luciferase reporter assay using promoters of the target genes and exogenous Nur77 expression. All the target promoters including Nur-RE-luc, StAR-luc, P450c17-luc, and 3*β*-HSD-luc were strongly repressed following overexpression of FOXA3 (Figure 5), suggesting that FOXA3 inhibits Nur77 transactivation. The repression of Nur77 transactivation may be through direct or indirect interaction of FOXA3 with Nur77.

### 3.6. FOXA3 Represses the Expression of *Nur77* and Its Target Steroidogenic Genes in Mouse Primary Leydig Cells

**Figure 6:**
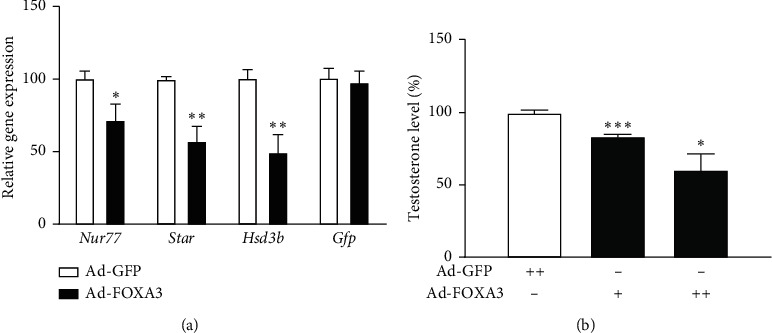
FOXA3 represses cAMP-induced expression of steroidogenic genes in primary Leydig cells. mRNA expression of steroidogenic genes in primary Leydig cells (A). Mouse primary Leydig cells were transduced with Ad-FOXA3 or Ad-GFP as control. After wash, the cells were further cultured for 48 h that included 4 h of cAMP (200 *μ*M) induction. The gene expression level was normalized to that of GAPDH. Testosterone production was significantly reduced by FOXA3 (B). Cells were transduced in a similar manner and maintained in 200 *μ*M of cAMP for 48 h Culture medium was harvested for RIA assay. The *t*-test was performed to analyze statistical significance. ^*∗*^*P* < 0.05; ^*∗∗*^*P* < 0.0; ^*∗∗∗*^*P* < 0.001.

FOXA3 represses the activity of the Nur77 promoter and thus of its target gene promoters (Figure 2). Furthermore, FOXA3 represses the transactivation of Nur77 (Figure 5), resulting in a dual repressive effect on Nur77 target promoters. To confirm the role of FOXA3 in testicular Leydig cells, we isolated primary Leydig cells from adult mice, infected them with FOXA3-expressing adenovirus (Ad-FOXA3), and analyzed the effect of FOXA3 on the expression of endogenous steroidogenic genes. Consistent with the results of the promoter-reporter assays in MA-10 cells, the expression of *Nur77* and steroidogenic genes including *StAR* and *Hsd3b* were significantly decreased when compared to that in cells infected with only GFP-expressing virus (Ad-GFP) (Figure 6(a)). These results suggest that FOXA3 represses the expression of steroidogenic genes in Leydig cells by reducing the expression of Nur77 and inhibiting Nur77 transactivation.

### 3.7. FOXA3 Modulates Steroidogenesis in Testicular Leydig Cells

Repression of steroidogenic gene expression by FOXA3 prompted us to evaluate the effect of FOXA3 on the production of testosterone in primary Leydig cells. We infected primary Leydig cells with either Ad-FOXA3 or Ad-GFP as a control. Following stimulation of the cells with cAMP for 48 h, media were collected and testosterone levels were analyzed. There was a significant reduction in the level of testosterone following Ad-FOXA3 infection compared to Ad-GFP control infection (Figure 6(b)). Together, these results suggest that FOXA3 modulates testicular steroidogenesis by controlling Nur77-induced expression of steroidogenic genes in Leydig cells.

## 4. Discussion

Testicular steroid production in Leydig cells is positively or negatively regulated by several signaling pathways and transcription factors [25, 26]. Among these, LH/cAMP stimulation is a major regulatory pathway for testicular steroidogenesis, which rapidly induces the expression of Nur77 to upregulate the expression of the steroid-producing genes [5]. In this study, we investigated the role of *Foxa3*, the only member of the *Foxa* subfamily that is expressed in the testis. *Foxa3* mRNA is expressed between postnatal days 6 and 70 during testicular development [16]. Its expression in the Leydig cells has major physiological importance compared to that in the spermatids as no histological difference was observed in the spermatids during the first wave of spermatogenesis in *Foxa3* null mice.

In our study, the repression of *Foxa3* during cAMP-induced expression of Nur77 in MA-10 Leydig cells suggests its repressive role during the steroidogenic process. This was evidenced by the decreased activity of Nur77 and its target steroidogenic gene promoters, following cAMP induction, when FOXA3 was overexpressed. CREB is a key transcription factor that regulates the expression of numerous genes including Nur77, during cellular development and differentiation [27, 28]. The decreased expression of steroidogenic genes following overexpression of FOXA3 may be due to inhibition of CREB transactivation as well as expression. In fact, previous studies revealed that the presence of FOXA binding sites near the CRE site in the promoter interferes with CREB binding and lower CRE activity [29]. However, we did not find any FOXA binding site close to CRE in the Nur77 promoter. Nevertheless, similar sequences were found in the vicinity, although these sequences were slightly different from the canonical FOXA binding sequences reported previously.

Garon et al. reported the regulation of the PDGFR-*α* promoter by FOXA3 in MLTC-1 Leydig cell lines by directly binding to its proximal recognition site [26]. Although they did not focus on the regulation of steroidogenic gene expression in their study, they reported that unlike the PDGFR-*α* promoter, FOXA3 overexpression tended to repress the promoter activity of genes known to be expressed in Leydig cells, including some steroidogenic genes such as *mCyp11a1*, *mStar*, and *mHsd3b1* [18]. In the present study, our results show that the expression of steroidogenic genes is repressed by FOXA3 through the inhibition of Nur77 expression and transactivation. However, we do not rule out the possibility of FOXA3-induced expression of PDGFR-*α* in MA-10 cells and its partial contribution to the repression of steroidogenic genes.

Primary Leydig cells infected with adenovirus expressing FOXA3 showed similar results as the MA-10 cell line. Moreover, this FOXA3-mediated repression of the steroidogenic genes translated to a reduced level of testicular testosterone. Because testosterone is a vital hormone for male reproduction, malfunction or altered expression of FOXA3 may affect male fertility. Reduced male fertility has been reported in *Foxa3* mutant mice when mated between 3 and 8 months of age [16].

## 5. Conclusion

Our findings suggest a repressive function of *Foxa3* during LH/cAMP-induced expression of Nur77 and Nur77-mediated testicular steroidogenesis in Leydig cells, resulting in a reduced level of testicular testosterone. Therefore, we postulate that FOXA3 functions as a modulator to maintain the balance of testicular testosterone by fine-tuning the expression of steroidogenic genes.

## Data Availability

The data used to support the findings of this study are available from the corresponding author upon request.

## Supplementary Materials

Supplementary Materials Supplementary Table 1. List of gene specific oligomers used for quantitative PCR.
