# Supplementary material for: FOXA3, a Negative Regulator of Nur77 Expression and Activity in Testicular Steroidogenesis
Source: Int J Endocrinol. 2021 Mar 3;2021:6619447. doi: 10.1155/2021/6619447 (PMC7946474; doi:10.1155/2021/6619447)
Supplement: Supplementary Materials — Supplementary Table 1. List of gene specific oligomers used for quantitative PCR. [file 6619447.f1.docx]

**List of gene specific oligomers used for Quantitative PCR**:

| 3β-HSD Sense | 5’-ACT GCA GGA GGT CAG AGC T-3’ |
| --- | --- |
| 3β-HSD Antisense | 5’-ATG GTC TGC CTG GGA ATG AC-3’ |
| β-actin Sense | 5’-TTC TAC AAT GAG CTG CGT GTG-3’ |
| β-actin Antisense | 5’-GGG GTG TTG AAG GTC TCA AA-3’ |
| CREB Sense | 5’ -TGA AGA AGC AGC ACG GAA GAG- 3’ |
| CREB Antisense | 5’ -GGT CCT TAA GTG CTT TTA GCT CCT- 3’ |
| FOXA3 Sense | 5’-CTG GGC TCA GTG AAG ATG GA-3’ |
| FOXA3 Antisense | 5’-CGG GAG GGT AGG GAG AGC-3’ |
| GAPDH Sense | 5’-ATC ACC ATC TTC CAG GAG CGA G-3’ |
| GAPDH Antisense | 5’-GAG ATG ATG ACC CTT TTG GCT CC-3’ |
| GFP Sense | 5’-GTC GTC CTG CTT CAT GTG G-3’ |
| GFP Antisense | 5’-GGC GAG GAG CTG TTC ACC-3’ |
| Nur77 Sense | 5’-CTC GCC ATC TAC ACC CAA CT-3’ |
| Nur77 Antisense | 5’-AGC CTT AGG CAA CTG CTC TG-3’ |
| P450c17 Sense | 5’-TGG CCC CCT TGC TCA TCC CA-3’ |
| P450c17 Antisense | 5’-TCG GGG ACC AGC TCC GAA GG-3’ |
| StAR Sense | 5’-TGT CAA GGA GAT CAA GGT CCT TG-3’ |
| StAR Antisense | 5’-CGA TAG GAC CTG GTT GAT GAT-3’ |
